# Perioperative blood glucose variability and autonomic nervous system activity in on-pump cardiac surgery patients: Study protocol of a single-center observational study

**DOI:** 10.1097/MD.0000000000031821

**Published:** 2022-11-25

**Authors:** Etienne Chazal, Anne-Laure Parmentier, Sebastien Pili-Floury, Malika Bouhaddi, Sophie Borot, Andrea Perrotti, Lucie Vettoretti, Julian Trajkovski, David Ferreira, Chloe Zanoni, Emmanuel Samain, Guillaume Besch, Lucie Salomon du Mont

**Affiliations:** a Department of Anesthesiology and Intensive Care Medicine, University Hospital of Besancon, and EA 3920, University of Franche-Comte, Besancon, France; b Clinical Methodology Center, INSERM CIC 1431, University Hospital of Besancon, and UMR 6249 Chrono Environment, University of Franche-Comte, Besancon, France; c Department of Physiology, Functional Investigations, University Hospital of Besancon, and EA 3920, University of Franche-Comte, Besancon, France; d Department of Endocrinology, Metabolism, Diabetes and Nutrition, University Hospital of Besancon, Besancon, France; e Department of Cardiac Surgery, University Hospital of Besancon, and EA 3920, University of Franche-Comte, Besancon, France; f Department of Anesthesiology and Intensive Care Medicine, University Hospital of Besancon, Besancon, France; g Department of Anesthesiology and Intensive Care Medicine, University Hospital of Besancon, and EA 481 Neuroscience, University of Bourgogne Franche-Comte, Besancon, France; h Department of Vascular Surgery University Hospital of Besancon, and EA 3920, University of Franche-Comte, Besancon, France.

**Keywords:** anesthesia, autonomous nervous system, blood glucose variability, cardiac surgery, endothelial function, inflammation, intensive care unit

## Abstract

On-pump coronary artery bypass graft (CABG) and surgical valve replacement (SVR) are high-risk procedures. Several studies reported that perioperative blood glucose (BG) variability was independently associated with impaired postoperative outcome. However, the underlying mechanisms contributing to increased perioperative BG variability and to its deleterious impact remain unknown. The hypothesis of the study is that perioperative BG variability could be related to perioperative alteration of the autonomic nervous system (ANS) activity and to preoperative BG variability.

**Methods and analysis:**

We designed a prospective observational single-center study. Four groups of 30 patients will be studied: group 1, including insulin-requiring type 2 diabetic patients undergoing on-pump CABG; group 2, including non-insulin-requiring type 2 diabetic patients undergoing on-pump CABG; group 3, including non-diabetic patients undergoing aortic SVR; and group 4, including non-diabetic patient undergoing on-pump CABG. Preoperative (baseline) and postoperative BG variability will be quantified using the Abbott’s Freestyle Libre Pro sensor allowing for continuous subcutaneous BG monitoring. Preoperative (baseline) and postoperative ANS activity will be measured using noninvasive continuous heart rate monitoring (Mooky HR memory®). Blood level and urinary concentration of inflammatory and endothelial dysfunction biomarkers will be measured from blood and urinary samples at the end of the surgery and on postoperative day 1 and 2. The primary objective is to describe the relationship between baseline BG variability and postoperative BG variability. The secondary objectives are to describe the relationship: between baseline and postoperative BG variability according to the diabetes phenotype and to the type of surgery; between the ANS activity and the BG variability; and between postoperative BG variability and, urinary and blood biomarkers.

## 1. Introduction

Blood glucose (BG) variability in diabetic and non-diabetic patients is independently associated with increased morbidity and mortality in the intensive care unit and after cardiac events.^[1–3]^ In cardiac surgery patients, BG variability is reported to be associated with severe postoperative complications – leading to an increase length of intensive care unit (ICU) stay – regardless of the quality of BG control obtained during the perioperative period.^[4–6]^

Several hypotheses have been raised to explain the deleterious effect of BG variability observed in diabetic patients. Basal state BG variability seems to depend on the diabetes phenotype and to differ between people with or without diabetes.^[7,8]^ Furthermore, some studies suggest an association between BG variability and the activity of the autonomic nervous system (ANS).^[9–13]^ Finally, other studies highlight that the deleterious effect of BG variability includes increased oxidative stress, endothelial dysfunction, and cell apoptosis.^[14–16]^ However, no study has specifically addressed this issue in cardiac surgery patients and the underlying mechanisms contributing to increased perioperative BG variability and to its deleterious impact remain unclear.

The aim of this study is to investigate whether postoperative GV could be related to preoperative GV and/or to perioperative alteration of the ANS activity in diabetic and non-diabetic cardiac surgery patients and the relationship between postoperative GV and blood and urinary level of inflammation and endothelial dysfunction biomarkers.

## 2. Objectives

The primary objective is to describe the relationship between baseline (preoperative) and postoperative BG variability within 48 hours after surgery.

Secondary objectives are: to describe the relationship between baseline and postoperative BG variability within 48 hours after surgery according to the diabetic status and to the type of surgery; to describe the relationship between the preoperative activity of the ANS and the baseline BG variability; to describe the relationship between the preoperative activity of the ANS and the postoperative BG variability within 48 hours after surgery; to describe the relationship between postoperative BG variability within 48 hours after surgery and the postoperative activity of the ANS within 48 hours after surgery; to describe the relationship between postoperative BG variability within 48 hours after surgery and the diabetes phenotype; to describe the relationship between postoperative BG variability within 48 hours after surgery and the urinary and blood level of biomarkers of inflammation and of endothelial dysfunction; and to describe the morbidity and the mortality within 30 days after surgery.

## 3. Methods and analysis

This manuscript is written in accordance with the SPIRIT guidelines for the reporting of interventional trial protocols.^[17]^

### 3.1. Trial design

This is a prospective observational single center study.

### 3.2. Eligibility

Adult patients who undergo planned on-pump coronary artery bypass surgery or aortic valve replacement surgery in the University Hospital of Besancon, France (Centre Hospitalier Universitaire (CHU) de Besancon) will be eligible for inclusion.

Four groups of 30 patients will be studied: group 1, including insulin-requiring type 2 diabetic patients undergoing on-pump coronary artery bypass graft (CABG); group 2, including non-insulin-requiring type 2 diabetic patients undergoing on-pump CABG; group 3, including non-diabetic patients undergoing aortic surgical valve replacement; and group 4, including non-diabetic patient undergoing on-pump CABG.

Inclusion and exclusion criteria for the present study are listed below.

### 3.3. Inclusion criteria

- Signed informed consent.
- Planned on-pump CABG or aortic valve replacement surgery (see above).
- Patient affiliated to French Social Security or equivalent.
- Ability to speak, write and understand French language.

### 3.4. Exclusion *criteria*

- Emergent surgery defined by a surgery performed < 48 hours after the anesthesia consultation.
- Combined surgery (coronary artery bypass combined with valve surgery, multiple valve replacement, ascending aorta surgery).
- Off-pump CABG.
- Type 1 diabetes mellitus.
- Chronic preoperative heart rhythm disorder (regular extrasystoles, chronic atrial fibrillation).
- Pacemaker dependence.
- Body mass index < 18 kg m^–2^ or >35 kg m^–2^.
- Age under 18 years or over 80 years.
- Pregnant or breast^-^feeding women.
- Expected life expectancy < 48 hours.
- Legal incapacity or limited legal capacity.
- Subjects without health insurance.
- Subjects within the exclusion period of another study.

### 3.5. Study procedure and patient management

Eligible patients will be screened and informed during the anesthesia consultation. Written informed consent will be obtained the same day by investigators after an oral explanation of the study.

After inclusion in the study, patient characteristics, past medical history including ASA physical status and Euroscore value, and preoperative fasting BG and glycosylated hemoglobin values will be collected.

All patients will be admitted to the Cardiac Surgery Department the day before surgery. All patients included in the study will be managed according to the current practices for the management of patients undergoing planned cardiac surgery. Briefly, on arrival in the operating room, standard monitoring (Carescape B850 monitor, General Electric Healthcare, USA) will be set up. A dedicated peripheral venous catheter will be inserted in a forearm vein to infuse anesthetic drugs. All patients will receive manually driven target^-^controlled infusion of propofol and remifentanil to maintain bispectral index (BIS) values between 40 and 60. All patients will be paralyzed using non depolarizing neuromuscular blocking agent (either cisatracurium or atracurium). After induction of anesthesia, patients will be intubated, and tidal volume and respiratory rate will be set to maintain the end^-^tidal carbon dioxide between 35 and 40 mm Hg. Inspired oxygen fraction will be adjusted to obtain a pulse oximetry ≥ 95%. A central venous catheter inserted in the internal jugular vein, a urinary catheter and an indwelling arterial catheter introduced in a radial artery will be implemented. No intravenous glucose will be administered intraoperatively in the absence of hypoglycemia (defined as <75 mg/dL [4.1 mmol/L]). Tranexamic acid (1.5 g before incision and 1.0 g before surgical wound closure) and antibiotic prophylaxis with cefuroxime (1.50 g before skin incision, reinjection at priming of the extracorporeal circulation and then 0.75 g every 2 h until end of surgery) or linezolid 600 mg in case of allergy to cefuroxime will be initiated before incision.

Intraoperative data describing the surgical procedure and the anesthetic management will be obtained from the computerized anesthesia monitoring sheet: type and duration of surgery; duration of aortic cross clamping; duration of extracorporeal circulation; intraoperative blood loss and urine output; intraoperative blood transfusion and total volume of fluids infused.

At the end of surgery, all patients will be admitted to the intensive care unit (ICU) and will stay during at least the first 48 postoperative hours, and then will be discharged to the ward. During the ICU stay, all patients will undergo the same standard monitoring (Carescape B850 monitor, General Electric Healthcare, USA) as in the operating room, that is the continuous monitoring of at least heart rate, electrocardiogram, pulse oximetry and invasive arterial pressure. Sedation will be stopped, and the patients will be extubated as soon as possible according to the usual criteria (i.e., free of sedation, normothermic, fulfilling specified respiratory criteria). Postoperative analgesia will consist in intravenous paracetamol 15 mg/kg 4 times a day and patient^-^controlled analgesia with morphine. Intravenous glucose infusion will be administered at a constant rate of 4.0 to 4.5 g/hours until oral feeding could be resumed and at least during the first 48 postoperative hours. BG control will be managed according to a validated and published dynamic insulin therapy protocol,^[18]^ adapted from Goldberg *et al*^[19]^ and implemented in our ICU since December 2005. Briefly, hourly BG measurement starts with the skin incision. All BG values are obtained, from glucose meter readings (Optium Xceed; Abbott Diabetes Care, UK) measured from arterial blood samples taken from the arterial line. The target BG level is 100 to 139 mg/dL (5.5–7.7 mmol/L). Intravenous insulin infusion starts with the first BG value > 139 mg/dl (7.7 mmol/L). After an initial bolus of insulin based on the BG value, the infusion rate is adjusted based on 3 parameters: the current BG value; the previous BG value; and the current insulin infusion rate. When hypoglycemia occurs, the insulin infusion is stopped and intravenous dextrose 30% is administered (10 mL if BG is between 50 and 74 mg/dL [2.7–4.1 mmol/L] and the patient is asymptomatic; 20 mL if BG is between 50 and 74 mg/dL [2.7–4.1 mmol/L] and the patient is symptomatic or if BG is <50 mg/dL [<2.7 mmol/L]). The insulin infusion is resumed 1 hour after the first BG value > 100 mg/dL (>5.5 mmol/L) at a lower rate (75% of the original rate if BG is between 50 and 74 mg/dL [2.7–4.1 mmol/L] and 50% of the original rate if BG is <50 mg/dL [<2.7 mmol/L]). Depending on the patient’s clinical condition, oral intakes are initiated as soon as possible, and the intravenous insulin infusion is replaced by subcutaneous insulin when necessary.

Post^-^operative morbidity including major adverse events (MAEs) (cardiac arrest, stroke, cardiac arrhythmia requiring therapeutic intervention, surgical wound infection, pneumonia, renal failure requiring dialysis, sepsis, surgical revision, readmission in the intensive care unit or in hospital) and post^-^operative mortality will be assessed on day 30 after surgery.

All data will be collected using the Clean Web web^-^based system (Telemedicine Technologies, France).

### 3.6. Assessment of perioperative blood glucose variability

After written informed consent is obtained, a subcutaneous glucose sensor Abbott® FreeStyle Libre Pro will be applied in the fatty part of the back of the upper arm of included patients, as recommended by the manufacturer. The Abbot® FreeStyle Libre Pro glucose sensor will allow to measure the baseline BG variability during at least 2 consecutive days prior to surgery using the following parameters: the standard deviation (SD) of BG, the coefficient of glycemic variability (CV) (defined as the ratio of the SD on the mean BG expressed as a percentage), the mean of the daily differences between the higher and the lower values of BG (MAGE), the glycemic lability index and the glycemic penalty index. In case of accidental removal of the sensor during this period or delayed surgery > 14 days after the inclusion, patients will be informed to bring back the sensor the day before surgery so that all blood glucose measurements stored in the sensor can be collected.

The subcutaneous glucose sensor Abbott® FreeStyle Libre Pro will be changed the day before surgery and applied in the same area to measure postoperative BG variability up to 7 days after surgery using the same parameters as preoperatively.

### 3.7. Assessment of the activity of the autonomous nervous system

The baseline activity of the ANS will be measured using the COMposite Autonomic Symptom Score (COMPASS) 31 questionnaire, completed the day before surgery, and using the continuous noninvasive^-^ heart rate sensor (MooKy HR memory®) applied on the chest wall the same day. The COMPASS 31 questionnaire is a validated test composed of 31 items assessing the clinical symptoms of the activity of the ANS divided into 6 domains: orthostatic intolerance (4 questions), vasomotor (3 questions), secretomotor (4 questions), gastrointestinal (12 questions), bladder (3 questions), and pupillomotor (5 questions).^[20]^ The COMPASS 31 score ranges from 0 to 100, higher score indicating more severe alteration of the ANS.^[20–22]^ The noninvasive^-^ heart rate monitoring using the MooKy HR memory® sensor will allow to quantify the activity of the NAS using the following heart rate variability metrics: the relative power of the low^-^frequency spectral band of the heart variability (LF) that is indicative of the sympathetic activity, the relative power of the high^-^frequency spectral band of the heart rate variability (HF), the ratio of LH^-^to^-^HF power (LF/HF) that is indicative of sympathetic to parasympathetic autonomic balance, and the the root mean square of successive differences between normal heartbeats (RMSSD) that is indicative of the parasympathetic activity.^[23–25]^

The MooKy HR memory® sensor will be temporarily removed during the surgery to allow the access to the surgical site. Then, the MooKy HR memory® sensor will be applied on the chest wall after admission in the ICU up to 48 hours after surgery. The postoperative activity of the ANS will be measured using the MooKy HR memory® using the same heart rate variability metrics as preoperatively. All heart rate and invasive arterial pressure values collected during the first 48 hours using the standard monitoring will be extracted from the Carescape B850 monitor and stored on a dedicated computer. The analysis of the data collected using the aHRV Analysis software (Nevrokard Izola, Slovenia) will allow the measurement of the postoperative activity of the ANS by calculating the following parameters: the standard deviation of the NN intervals (SDNN), the standard deviation of the average NN intervals for each 5 minutes segment of a 24 hours recording (SDANN), the percentage of successive RR intervals that differ by more than 50 ms (pNN50), the LF, the HF, the LF/HF and the RMSSD. SDNN, SDANN and pNN50 are indicators of the global activity of the ANS. Morever, the aHRV Analysis software (Nevrokard Izola, Slovenia) will allow to quantify the activity of the baroreflex by combining the temporal trend of the heart rate and of the invasive arterial pressure values collected using the Carescape B850 monitor within 48 hours after surgery.

### 3.8. Outcome measures

The primary outcome will be the mean difference between the preoperative (baseline) and the postoperative CV, and its 95% confidence interval, measured using the subcutaneous glucose sensor Abbott® Free Style Libre Pro.

Secondary outcomes will: the preoperative and postoperative BG variability, quantified by the preoperative and postoperative values of: the SD of BG, the CV, the MAGE, the glycemic lability index and the glycemic penalty index; the preoperative and postoperative activity of the ANS, quantified by the preoperative and postoperative values of: the COMPASS 31 score, the LF, the HF, the LF/HF ratio, the RMSSD, the SDNN, the SDANN, and the pNN50; the diabetes phenotype, described by the preoperative fasting BG and glycosylated hemoglobin values; the blood and urinary levels of biomarkers of inflammation (IL^-^2, IL^-^6, IL^-^10, TNF^-^alpha, 8^-^iso^-^prostanglandin^-^F_2*α*_) and of endothelial function [Endocan, angiopoietin^-^1 (Ang^-^1), angiopoietin^-^2 (Ang^-^2), vascular endothelial (VE)^-^cadherin]; and postoperative morbidity and mortality at day 30 after surgery. Postoperative morbidity includes cardiac arrest, stroke, cardiac arrhythmia requiring therapeutic intervention, surgical wound infection, pneumonia, renal failure requiring dialysis, sepsis, surgical revision, readmission in the intensive care unit or in hospital.

### 3.9. Laboratory measurements

Blood and urinary levels of biomarkers of inflammation (IL-2, IL-6, IL-10, TNF-alpha, 8-iso^-^prostanglandin^-^F_2*α*_) and of endothelial function [Endocan, angiopoietin^-^1 (Ang^-^1), angiopoietin^-^2 (Ang^-^2), vascular endothelial (VE)^-^cadherin] will be measured from blood and urinary samples performed the day before surgery, on arrival in the ICU and at 24 hours and at 48 hours after admission in the ICU. Additional blood and/or urinary analysis will be performed if necessary, depending on the hypotheses formulated from the results of the study.

### 3.10. Safety

All serious adverse events will be collected and reviewed by the principal investigator and reported to the trial sponsor (CHU Besancon, Besancon, France). Study insurance has been contracted for all participants by the trial sponsor (CHU Besancon, Besancon, France).

The use of the MooKy HR memory® device and of the Abbott® FreeStyle Libre Pro subcutaneous glucose sensor comply with the CE marking and with the recommendation of the manufacturer.

### 3.11. Sample size calculation

Four groups of patients will be included to describe the relationship between preoperative (baseline) and early postoperative BG variability in diabetic (groups 1 and 2) and non^-^diabetic patients (groups 3 and 4), and between BG variability and the ANS activity in patients treated (groups 1, 2 and 4) or not (group 3) with beta^-^blockers: group 1, including patients with insulin^-^requiring type 2 diabetes mellitus undergoing planned on^-^pump CABG surgery; group 2, including patients with non^-^insulin^-^requiring type 2 diabetes mellitus undergoing planned on^-^pump CABG surgery: group 3, including non^-^diabetic patients undergoing planned aortic valve replacement surgery; and group 4, including non^-^diabetic patients undergoing planned on^-^pump CABG surgery.

No previous study has described the difference between the preoperative (baseline) and the early postoperative CV (primary outcome) in cardiac surgery patients. Thus, no assumption can be made on the expected value of the primary outcome. The sample size calculation was based in the expected measurement accuracy of the primary outcome in each group.

Considering an alpha risk at 0.05 and an estimated standard deviation of CV of 8.2 (basal state) (RE Pratley, 2018 Nov), we hypothesized that the 95% confidence interval range of the difference between the baseline and the early postoperative CV will be no more than 6.12 (conservative assumption considering that the variance will be lower because of the repeated measurement of CV in the same patients). A sample size of 30 patients per group was calculated using the PASS software (“Confidence Intervals for 1 Mean” method, Hahn, G.J. and Meeker, W.Q. 1991. Statistical intervals. John Wiley & Sons. New York).

Thus, the inclusion of a total sample size of 120 patients (30 patients per group) is planned.

### 3.12. Statistical analysis

Categorical variables will be compared using the Chi^-^square test or Fisher’s exact test. Normally distributed quantitative variables will be compared using the Student *t* test or an analysis of variance (ANOVA). Non^-^normally distributed quantitative variables will be analyzed using the non^-^parametric Wilcoxon or Kruskall^-^Wallis tests. Statistical tests for repeated measures will be used for before^-^ after comparisons (paired Student *t* test or paired Wilcoxon test, 1^-^way ANOVA with repeated measures or Friedman test).

The primary outcome will be described using the mean and the 95% confidence interval of the mean. The temporal trend of preoperative, intraoperative, and postoperative BG variability will be described by the mean and the 95% confidence interval of the mean of each variable describing BG variability and figured as boxplot. The relationship between preoperative and postoperative BG variability, between BG variability and the ANS activity and between the postoperative BG variability and the blood and urinary level of biomarkers of inflammation and endothelial function will be investigated using the Pearson or the Spearman correlation coefficient and univariate linear regression models and presented on adequate figures.

### 3.13. Monitoring

The Department of Research and Clinical Investigation of our institution will monitor all written informed consent and check inclusion and exclusion criteria.

## 4. Ethics and dissemination

### 4.1. Ethic approval and registration

This study was approved by the French Ethics Committee (Comité de Protection des Personnes Ile de France IV, Hopital Saint Louis, Assistance Publique des Hôpitaux de Paris, Chairperson Dr Julien Dumurgier, n° 2021/616 on January 418, 2022). The study is registered on ClinicalTrials.gov (Identifier: NCT05454735, principal investigator: Prof Guillaume Besch, date of registration: July 12, 2022). This study is conducted in accordance with GCP-ICH-6^[26,27]^ in a single university affiliated hospital (CHU de Besancon, Besancon, France). Eligible patients will be screened and informed during the anesthesia consultation. Written informed consent will be obtained by investigators prior to inclusion the day before surgery.

### 4.2. Planning and dissemination

Inclusion will start on October 2022. The estimated duration of recruitment is 12 months. The university hospital of Besancon (CHU Besancon, Besancon, France) is the trial sponsor and the holder of all data and publication rights. The results of the study will be submitted for publication in a peer-review international medical journal and presented in abstract form in national and international conferences.

## Author contributions

^--^All authors approved the final version of the manuscript to be published and are accountable for all aspects of the work.

**Conceptualization:** Lucie Salomon du Mont, Guillaume Besch, Anne-Laure Parmentier, Etienne Chazal, Sophie Borot, Malika Bouhaddi, Sebastien Pili-Floury.

**Data curation:** Lucie Salomon du Mont, Guillaume Besch, Etienne Chazal, Sebastien Pili^-^Floury.

**Formal analysis:** Lucie Salomon du Mont, Guillaume Besch, Etienne Chazal, Anne^-^Laure Parmentier, David Ferreira, Chloe Zanoni, Andrea Perrotti, Sophie Borot, Malika Bouhaddi, Emmanuel Samain, Sebastien Pili^-^Floury.

**Funding acquisition:** Guillaume Besch, Etienne Chazal, Anne^-^Laure Parmentier.

**Investigation:** Lucie Salomon du Mont, Guillaume Besch, Etienne Chazal, Lucie Vettoretti, Julian Trajkovski, Sebastien Pili^-^Floury, Chloe Zanoni, David Ferreira.

**Methodology:** Guillaume Besch, Anne^-^Laure Parmentier, Etienne Chazal, Malika Bouhaddi, Sophie Borot.

**Resources:** Guillaume Besch, Sebastien Pili^-^Floury, Lucie Vettoretti.

**Supervision:** Lucie Salomon du Mont, Guillaume Besch, Etienne Chazal, Sebastien Pili^-^Floury.

**Validation:** Lucie Salomon du Mont, Guillaume Besch, Etienne Chazal, Anne^-^Laure Parmentier, Sophie Borot, Malika Bouhaddi, David Ferreira, Andrea Perrotti, Emmanuel Samain, Sebastien Pili^-^Floury, Chloe Zanoni.

**Visualization:** Lucie Salomon du Mont, Guillaume Besch, Etienne Chazal, Anne^-^Laure Parmentier, Sophie Borot, Malika Bouhaddi, David Ferreira, Andrea Perrotti, Emmanuel Samain, Sebastien Pili^-^Floury, Chloe Zanoni.

**Writing – original draft:** Lucie Salomon du Mont, Guillaume Besch, Etienne Chazal.

**Writing – review & editing:** Lucie Salomon du Mont, Guillaume Besch, Etienne Chazal, Anne^-^Laure Parmentier, Julian Trajkovski, David Ferreira, Chloe Zanoni, Andrea Perrotti, Sophie Borot, Malika Bouhaddi, Emmanuel Samain, Sebastien Pili^-^Floury.

## References

[1] Egi M Bellomo R Stachowski E. Variability of blood glucose concentration and short^-^term mortality in critically ill patients. Anesthesiology. 2006;105:244–52. 16871057 10.1097/00000542-200608000-00006

[2] Besch G Pili-Floury S Morel C. Impact of post^-^procedural glycemic variability on cardiovascular morbidity and mortality after transcatheter aortic valve implantation: a post hoc cohort analysis. Cardiovasc Diabetol. 2019;18. 10.1186/s12933-019-0831-3 PMC6410509 30857532

[3] Zhang J He L Cao S. Effect of glycemic variability on short term prognosis in acute myocardial infarction subjects undergoing primary percutaneous coronary interventions. Diabetol Metab Syndr. 2014;6:76. 25013458 10.1186/1758-5996-6-76 PMC4091650

[4] Ogawa S Okawa Y Sawada K. Continuous postoperative insulin infusion reduces deep sternal wound infection in patients with diabetes undergoing coronary artery bypass grafting using bilateral internal mammary artery grafts: a propensity^-^matched analysis. Eur J Cardiothorac Surg. 2016;49:420–6. 25825261 10.1093/ejcts/ezv106

[5] Subramaniam 2014 Increased Glycemic Variability in Patients With El.pdf.

[6] Bansal B Carvalho P Mehta Y. Prognostic significance of glycemic variability after cardiac surgery. J Diabetes Complicat. 2016;30:613–7. 10.1016/j.jdiacomp.2016.02.010 26965795

[7] Lin R Brown F James S. Continuous glucose monitoring: a review of the evidence in type 1 and 2 diabetes mellitus. Diabet Med. 2021;38. 10.1111/dme.14528 33496979

[8] Smith-Palmer J Brändle M Trevisan R. Assessment of the association between glycemic variability and diabetes^-^related complications in type 1 and type 2 diabetes. Diabetes Res Clin Pract. 2014;105:273–84. 25023992 10.1016/j.diabres.2014.06.007

[9] Fleischer J Lebech Cichosz S Hoeyem P. Glycemic variability is associated with reduced cardiac autonomic modulation in women with type 2 diabetes. Diabetes Care. 2015:dc140654. 10.2337/dc14-0654 25573884

[10] Jun JE Jin SM Baek J. The association between glycemic variability and diabetic cardiovascular autonomic neuropathy in patients with type 2 diabetes. Cardiovasc Diabetol. 2015;14:70. 26041130 10.1186/s12933-015-0233-0 PMC4462181

[11] Helleputte S De Backer T Lapauw B. The relationship between glycaemic variability and cardiovascular autonomic dysfunction in patients with type 1 diabetes: a systematic review. Diabetes Metab Res Rev. 2020;36. 10.1002/dmrr.3301 32073212

[12] Matsutani D Sakamoto M Iuchi H. Glycemic variability in continuous glucose monitoring is inversely associated with baroreflex sensitivity in type 2 diabetes: a preliminary report. Cardiovasc Diabetol. 2018;17:36. 29514695 10.1186/s12933-018-0683-2 PMC5840775

[13] Xu W Zhu Y Yang X. Glycemic variability is an important risk factor for cardiovascular autonomic neuropathy in newly diagnosed type 2 diabetic patients. Int J Cardiol. 2016;215:263–8. 27128543 10.1016/j.ijcard.2016.04.078

[14] Monnier. Activation of Oxidative Stress by Acute Glucose Fl.pdf.

[15] Ceriello. 2019 Glycaemic variability in diabetes clinical and th.pdf. 10.1016/S2213-8587(18)30136-0 30115599

[16] Buscemi S Re A Batsis JA. Glycaemic variability using continuous glucose monitoring and endothelial function in the metabolic syndrome and in Type 2 diabetes: glycaemic variability and endothelial function. Diabet Med. 2010;27:872–8. 20653743 10.1111/j.1464-5491.2010.03059.x

[17] Chan AW Tetzlaff JM Altman DG. SPIRIT 2013 statement: defining standard protocol items for clinical trials. Ann Intern Med. 2013;158:200. 23295957 10.7326/0003-4819-158-3-201302050-00583 PMC5114123

[18] Studer C Sankou W Penfornis A. Efficacy and safety of an insulin infusion protocol during and after cardiac surgery. Diabetes Metab. 2010;36:71–8. 20097589 10.1016/j.diabet.2009.05.008

[19] Goldberg PA Siegel MD Sherwin RS. Implementation of a safe and effective insulin infusion protocol in a medical intensive care unit. Diabetes Care. 2004;27:461–7. 14747229 10.2337/diacare.27.2.461

[20] Sletten DM Suarez GA Low PA. COMPASS 31: a refined and abbreviated composite autonomic symptom score. Mayo Clin Proc. 2012;87:1196–201. 23218087 10.1016/j.mayocp.2012.10.013 PMC3541923

[21] D’Amato C Greco C Lombardo G. The diagnostic usefulness of the combined COMPASS 31 questionnaire and electrochemical skin conductance for diabetic cardiovascular autonomic neuropathy and diabetic polyneuropathy. J Peripher Nerv Syst JPNS. 2020;25:44–53. 31985124 10.1111/jns.12366

[22] Greco C Di Gennaro F D’Amato C. Validation of the composite autonomic symptom score 31 (COMPASS 31) for the assessment of symptoms of autonomic neuropathy in people with diabetes. Diabet Med J Br Diabet Assoc. 2017;34:834–8. 10.1111/dme.13310 27990686

[23] Heart rate variability: standards of measurement, physiological interpretation, and clinical use. Task force of the European society of cardiology and the North American Society of Pacing and Electrophysiology. Eur Heart J. 1996;17:354–81. 8737210

[24] Akselrod S Gordon D Ubel FA. Power spectrum analysis of heart rate fluctuation: a quantitative probe of beat^-^to^-^beat cardiovascular control. Science. 1981;213:220–2. 6166045 10.1126/science.6166045

[25] Benichou T Pereira B Mermillod M. Heart rate variability in type 2 diabetes mellitus: a systematic review and meta^-^analysis. PLoS One. 2018;13:e0195166. 29608603 10.1371/journal.pone.0195166 PMC5880391

[26] Toulouse E Lafont B Granier S. French legal approach to patient consent in clinical research. Anaesth Crit Care Pain Med. 2020;39:883–5. 33130015 10.1016/j.accpm.2020.10.012

[27] Toulouse E Masseguin C Lafont B. French legal approach to clinical research. Anaesth Crit Care Pain Med. 2018;37:607–14. 30580775 10.1016/j.accpm.2018.10.013

